# Phylogenomic tree of Cercozoa based on single-cell transcriptomes from 100 uncultured cells

**DOI:** 10.1186/s12915-026-02536-4

**Published:** 2026-01-30

**Authors:** Gordon Lax, Elizabeth C. Cooney, Vasily Zlatogursky, Mahara Mtawali, Noriko Okamoto, Victoria K. L. Jacko-Reynolds, Saelin Bjornson, Corey Holt, Vedprakash G. Hurdeal, Daniele Giannotti, Patrick J. Keeling

**Affiliations:** 1 Department of Botany, University of British Columbia, Vancouver, Canada; 2 Department of Biology, Dalhousie University, Halifax, Canada; 3 Hakai Institute, Heriot Bay, Canada; 4 Department of Organismal Biology, Uppsala University, Uppsala, Sweden; 5 School of Biosciences, University of Melbourne, Melbourne, Australia; 6 Department of Life Sciences, University of Bath, Claverton Down, Bath, UK

**Keywords:** Multigene, Rhizaria, RNA-seq, Protist, Phaeodaria, *Cercomonas*

## Abstract

**Background:**

Cercozoa are single-celled eukaryotes (protists) and are part of the supergroup Rhizaria. Cercozoans have vastly different morphologies and are defined by their phylogenetic affinity. While the group includes some well-known and well-researched taxa, like the chlorarachniophytes, we know very little about the remainder. Most of these are predatory protists found in soil and marine sediments, but they also include marine plankton and are underrepresented in multigene phylogenetic trees of Rhizaria, thus missing much of their diversity. We employed single-cell transcriptomics to broadly sample this uncultured diversity of Cercozoa.

**Results:**

We generated a taxon-comprehensive multigene tree of Cercozoa that includes many previously unsampled groups, increasing taxon sampling by more than 300%. We report five novel and previously unknown lineages and two lineages that were known only from environmental sequences. Several previously established clades are recovered, like Thecofilosea, Phaeodaria, and Thaumatomonadida, but others, like the class Imbricatea, are not. We find both single and double amino-acid insertions between polyubiquitin monomers in all our assemblies, suggesting a complex pattern across Cercozoa.

**Conclusions:**

A single-cell transcriptomics approach generated a wealth of molecular and morphological image data for phylogenomics. This phylogenetic framework is in turn the groundwork for additional analyses to further our understanding of the basic biology of Cercozoa and their diversity. This study also highlights the number of previously unsampled taxa and completely novel lineages in Rhizaria and Cercozoa in particular.

**Supplementary Information:**

The online version contains supplementary material available at 10.1186/s12915-026-02536-4.

## Background

Cercozoa is a major lineage of microbial eukaryotes that is abundant across a wide range of ecosystems, has significant and diverse ecological impacts, and is highly diverse at both molecular and morphological levels [1–4]. Unlike other major lineages of eukaryotes, the phylum Cercozoa was only circumscribed through molecular phylogeny: the group lacks any shared morphology or a common body plan, so its monophyly was never recognized before molecular gene trees showed them to be related [5–7]. Cercozoan morphological diversity is extreme, with a wide range of unique characters and body plans ranging from small flagellates and amoeboflagellates, to naked and shelled testate amoebae, and to heliozoan-like amoebae and massive planktonic predators with mineralized endoskeletons [8–10]. Its ecological diversity is equally vast, with predators, grazers, phototrophs, and parasites common and abundant in freshwater, terrestrial, coastal, and deep-sea environments [1, 11, 12].

How this diversity arose and how their many unique characteristics evolved are interesting questions, but we have few insights because Cercozoa are also one of the most poorly studied of all eukaryotic groups. As a consequence, there are sparse genomic resources (a single genome and a few transcriptomes outside the chlorarachniophytes (Chlorarachnea) [13]), relatively few formally described species given the size of the group, and no well-sampled and strongly supported phylogeny, since for most lineages only a single gene, the small subunit of ribosomal RNA (SSU rRNA), is available.

Understanding the diversity and evolution of Cercozoa requires a well-supported phylogenetic tree, and while the breadth of taxa in the current SSU phylogenies has grown rapidly, many major subgroups in the tree are not well-supported (e.g. [14]). In addition, some groups encode highly divergent SSU rRNA genes, including some of the small flagellates like *Helkesimastix*, or the giant deep-sea phaeodarians (Phaeodaria), which drastically differ from other cercozoans in morphology and are superficially more similar to another group, the Radiolaria, to the extent that they were once classified as such and have since been reassigned [15, 16]. In addition, although Cercozoa are abundant in a wide range of global ecosystems, most of the current data come from terrestrial taxa [1, 2, 17], whereas marine environments are under-sampled [3, 4].

New cultures of cercozoans are established at a low but steady rate [3, 18–22], but even in these cases, often the SSU rRNA gene represents the only available molecular data, so the current genomic data are sparse and biased. Evidence of this can also be seen in the many cercozoan SSU rRNA clades that remain solely made up of environmental sequences [23]. Many of these were discovered over 20 years ago but still remain essentially uncharacterized.

Here, we have used a single-cell transcriptome approach [24–26] to circumvent the lack of cultured representatives and establish a strong molecular foundation for interpreting the diversity of Cercozoa. Specifically, we generated 119 single-cell transcriptomes from diverse cells broadly representing most of the clades across the cercozoan tree, emphasizing underrepresented groups and habitats, like marine ecosystems. We provide the first substantial sampling from two large and complex groups that are morphologically unlike other cercozoans and more reminiscent of other major groups: the phaeodarians (which resemble Radiolaria) and the Desmothoracida (which resemble heliozoans). Using these 119 single-cell transcriptomes, we have generated a taxon-rich multigene analysis based on 70 genes from 115 taxa. The tree highlights cercozoan diversity and is the first multigene tree to sample most known subgroups. Comparing this phylogenetic tree to existing trees based on SSU rRNA, we confirm some of the well-known clades, like Thecofilosea, whereas other proposed taxa, like Imbricatea, are shown to be polyphyletic. Several cells fall into clades that were previously known only from environmental sequences, and nine cells represent four novel clades that have never been sampled, even in environmental rRNA studies. Overall, this work begins to provide a well-supported backbone on which to begin to infer the evolution of a major but often overlooked group of eukaryotes.

## Results and Discussion

### Isolating diverse Cercozoan cells from nature

**Fig. 1:**
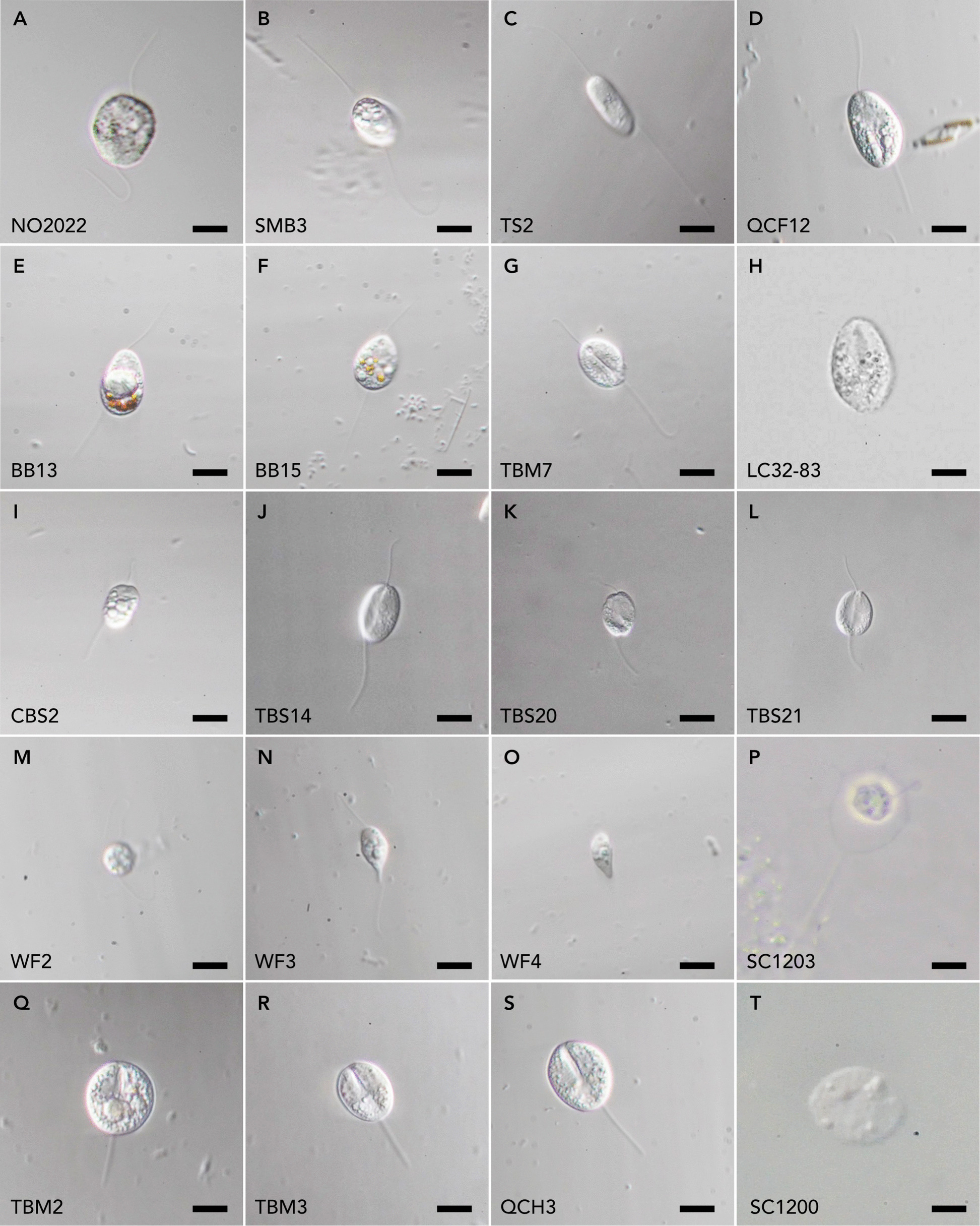
Light micrographs of representatives of novel cercozoan cells from which transcriptome data were obtained. This is a small fraction of the 119 cells analysed, chosen to show the overall morphological diversity of the cells we observed. Images of other cells are available in Additional Files 1 and 2: Figs. S1 and S2. Scale bars for **A**, **B**, **C**, **D**, **E**, **F**, **G, H**, **I**, **J**, **K**, **L**, **M**, **N**, **O**, **P**, **Q**, **R**, and **S** are 10 µm and 20 µm for **T**

We collected 119 cells preliminarily identified as likely being cercozoans (in itself a challenging task due to their morphological variation) from 40 different locations over a span of 5 years (see Additional File 5: Table S1 for details). From each of these cells, cDNA and library construction were carried out, leading to 99 cells that yielded relatively high-quality transcriptome data based on recovery of phylogenomic marker genes. Some morphotypes determined to be the same species were co-assembled at the marker-gene level in PhyloFisher, yielding novel multigene data for 70 discrete taxa in our final dataset. These cells represent an incredible diversity in morphology (see Fig. 1 for representatives and Additional Files 1 & 2: Figs. S1 & S2 for additional micrographs). Most available cercozoan genomes and transcriptomes come from the photosynthetic chlorarachniophytes and the euglyphids, and all of them are from cultured species. The new transcriptomic data increase sampling across the whole of Cercozoa by 470% when considering all isolates, or more conservatively by almost 330% when only distinct taxa are considered.

From the transcriptomes, the SSU rRNA was first extracted to get a rough idea of the identity of each cell and to seek evidence for cases where the species might already have been described formally (some transcriptomes did not have any SSU rRNA sequences recovered). The SSU rRNA tree (Additional File 3: Fig. S3) showed that the sampling does indeed cover most known cercozoan subgroups, with a particularly strong representation from thecofilosans, a very large, diverse, and under-sampled subgroup. Thirty cells were sufficiently closely related to known species, and 32 were sufficiently closely related to known genera, to be given those names, while 45 lacked close, described relatives.

### Overall structure of the phylogenomic tree

**Fig. 2:**
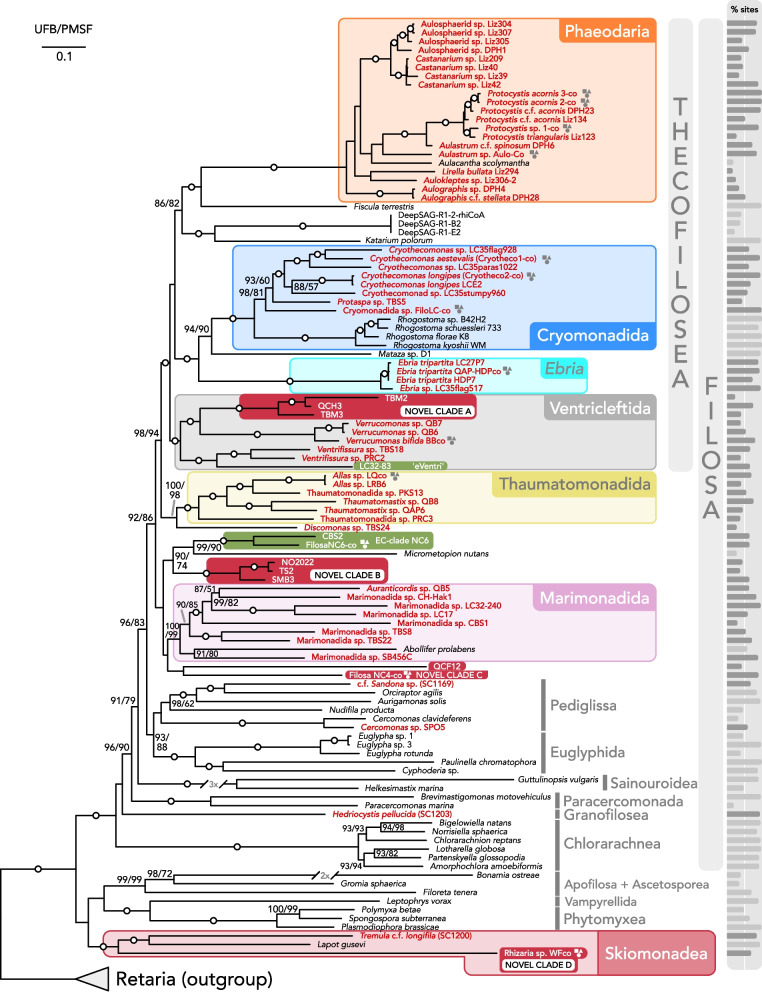
Multigene tree of Cercozoa based on 70 genes (12,261 amino acid characters) and 115 taxa, generated with Maximum Likelihood under the LG + C60 + G model with 1000 ultrafast bootstraps (UFB) and 200 non-parametric bootstraps (PMSF). Full bootstrap support in both analyses (100%) is denoted by a circle on the corresponding branch, while support values under 80% are not shown. Taxa characterized in this study are marked in red (or white) and bold, novel clades have a red box around them, and previously reported environmental clades have a green box around them. Co-assemblies of single-cell transcriptomes are denoted with a circle-square-triangle symbol. Bars on the right depict percentage of sites recovered for each taxon for the 70-gene dataset (Additional File 7: Table S3)

Thecofilosea ancestrally possess an extracellular organic theca and are one of the most diverse and widespread subgroups of Cercozoa but have also been historically under-sampled, except for a few taxa (e.g. *Cryothecomonas*). Our data confirms the deep divergence and monophyly of Thecofilosea (Fig. 2), with Phaeodaria, Cryomonadida, and Ventricleftida (ventricleftids) being major subgroups. In addition, many other flagellated and amoeboid gliding and surface-associated taxa like *Ebria* and *Katarium* all fall within Thecofilosea with strong support. Except for phaeodarians and *Ebria*, which are likely fully planktonic, all other thecofilosan cells appear to be at least partially surface-associated at some point in their life cycle. *Cryothecomonas*, for example, is known to attach to and be parasitic on diatoms [27], and we also found evidence for the latter being more widespread within the group. For example, all three cells used for co-assembly Cryotheco1-co were isolated from inside different diatom frustules. The taxonomic delineation between *Protaspa* and *Cryothecomonas* is currently unclear [3, 4] and likely requires additional sampling for molecular sequencing and ultrastructural studies since they are morphologically very similar.

Interestingly, the genus *Ebria* falls in the same place as it does in the SSU rRNA tree, sister to a clade composed of Cryomonadida plus *Mataza*, all within Thecofilosea [3, 28]. The closest relative to *Ebria* in SSU rRNA phylogenies is *Botuliforma*—a thecate benthic amoeboflagellate isolated from anoxic marine sediment that has been sequenced and reported only once [4]. This is in stark contrast to *Ebria*, which is a marine planktonic flagellate with an internal siliceous skeleton [29]. Very little is known about the life cycle and evolution of *Ebria*, and acquiring genomic or transcriptomic data from *Botuliforma* would be a crucial step in understanding the evolutionary history of *Ebria*, particularly since some members of the Thecofilosea have been shown to share ultrastructural traits [30], but such data is very limited for *Ebria* [29] or absent for *Botuliforma*. *Hermesinum*, which is morphologically very similar to *Ebria*, has also been shown to be closely related to the latter based on SSU rRNA analyses, so genomic data from this genus might also shed light on this evolution (Additional File 3: Fig. S3) [28].

Two other previously proposed cercozoan subgroups that are recovered with high support are Marimonadida and Thaumatomonadida (Fig. 2) [3]. Members of Marimonadida are flagellated swimmers and gliders and have no scales, whereas flagellated Thaumatomonadida possess scales [31, 32]. The tree supports the monophyly of several genera originally included in Marimonadida, such as *Pseudopirsonia*, *Auranticordis*, and *Abollifer*, as well as several cells of unknown genera, like the co-assembly SB456C. Thaumatomonadida are similarly made up of several of the genera originally proposed to be in this group: *Thaumatomastix*, *Allas*, and *Discomonas* [3, 33].

We do not recover a monophyletic group corresponding to the Sarcomonadea (Fig. 2 [3, 31]), but do recover both Pediglissa and Paracercomonada, which are the two orders that were proposed to make up Sarcomonadea [31]. Pediglissa includes cercomonads (Cercomonadida) and Glissomonadida (*Sandona* sp. SC1169); both groups are composed of bi-flagellated naked posterior gliders with an amoeboid feeding stage [31], and while the support for this group is low in our phylogeny at 70/79% (UFB/PMSF), there is better support (93/88%) for a group including Pediglissa and euglyphids. The cells *Cercomonas* SPO5 and *Sandona* SC1169 both branch with members of the Cercomonadida, but Paracercomonada branches outside this group, rendering Sarcomonadea non-monophyletic, something that has been reported previously [3, 31] and further complicating the taxonomic definition of the group. Cavalier-Smith et al. placed *Paracercomonas* in a separate subclass Paracercomonada from other Cercomonadida [31], but kept the taxon Sarcomonadea despite recovering it as paraphyletic.

More strikingly, we do not recover any support for the Imbricatea, which was proposed based on SSU rRNA phylogenies [3, 34] and contains few consistent morphological synapomorphies other than potentially tubular mitochondrial cristae [31, 32]. We find various lineages that were proposed to be members of this group to be polyphyletic, in many cases with strong support. This is congruent with a study by Cavalier-Smith et al., which shows Imbricatea to be non-monophyletic in a smaller multigene phylogeny [31].

### Phaeodaria

Phaeodarians are large, planktonic, amoeboid heterotrophs that are most common in the deep ocean and were long thought to be members of Radiolaria due to the overall similarities occurring between them as large amoebae with complex internal mineralized skeletons [15, 35, 36]. However, the first SSU rRNA data from the group showed they were actually cercozoans, probably related to Thecofilosea, and the single small EST (expressed sequence tag) data set from one species, *Aulacantha scolymantha*, also showed this [15, 16, 35]. We have now sequenced transcriptomes from 20 phaeodarian cells, which confirms they branch within Thecofilosea (Fig. 2). While this is not a new placement, it is worth emphasizing this position since the previously available data was limited, and the new phaeodarian transcriptomes are among the best in our dataset (some co-assemblies reaching 99% completeness). Single-cell transcriptomics seems like an ideal way to approach these difficult-to-access deep sea cells for the future. Our sampling mostly focuses on the various aulosphaerids (e.g. *Aulastrum*), aulacanthids (*Aulokleptes*, *Aulographis*), and *Protocystis* and shows high support for the monophyly of *Protocystis* and *Castanarium*, but several other groups are not well represented and appear to be non-monophyletic (including aulosphaerids and aulacanthids), and the overall deep nodes in phaeodarian phylogeny are not yet resolved. A focused study on phaeodarians should allow for a larger matrix of more genes, and, if mixed with wider taxon sampling, a well-resolved phylogeny of the group may be possible.

### Novel and environmental clades

Bass et al. (2004) identified several new clades of Cercozoa based only on environmental sequencing and called them ‘Novel Clades (NC)’. Some of these have remained essentially uncharacterized even after 20 years, so we specifically examined any potential cases where an isolated cell represented these clades and found two cases. The morphologies of these cells appear similar to one another and to other cercozoans at first glance, but closer inspection suggests there are differences in morphology and behaviour.

In the first case, the SSU rRNA tree shows that cells CBS2 and co-assembly FilosaNC6-co (TBS14, TBS20, TBS21) correspond to Novel Clade 6 (NC6) (Additional File 3: Fig. S3). These cells are inconspicuous biflagellates; in the case of FilosaNC6, the cells are all dorsoventrally flattened, oval to oblong with notches on both the anterior and posterior ends, and move via a ‘bouncing’ motion on their posterior flagellum (Fig. 1J, K, L, Videos TBS14, TBS20, TBS21 — Borealis Data). Cell CBS2 exhibits a similar pattern of movement, but the cell body is less flattened, more oblong, and is full of granulated reflective inclusions (Fig. 1I). All cells ‘whip’ their anterior flagellum up and down (Videos CBS2 — Borealis Data). In the phylogenomic tree, this group branches with another novel clade discovered here and the genus *Micrometopion* (see below), with low to moderate support (90% UFB, 74% PMSF).

The second case is cell LC32-83, corresponding to the environmental clade dubbed ‘eVentri’ [14, 37], which is sister to *Ventrifissura* in our phylogenomic tree (Fig. 2), and in turn, these two are sister to *Verrucomonas* with full support. The ‘eVentri’ environmental data, as well as *Verrucomonas* and *Ventrifissura*, are all known from marine sediment [4, 14], but exact placement of ‘eVentri’ was unclear from SSU rRNA data. Altogether, we show that these taxa make up the proposed Ventricleftida [3] with full support, despite not being recovered in most SSU rRNA phylogenies [3, 14, 20]. Ventricleftids are amoeboid biflagellates with a theca but no scales [4, 32]. Our cell LC32-83 shares certain morphological characteristics with the ventricleftids *Ventrifissura velata* and *Ventrifissura artocarpoidea* (Fig. 1H; [4, 14]). All are biflagellated and have a ‘dimpled’ surface texture, although it is more pronounced on *V. velata* and *V. artocarpoidea*. It should be noted that many thecofilosans, including *Ventrifissura*, have life cycles that are poorly understood, with diverse morphologies [3, 14], so it remains possible that different life stages of related lineages are being observed, making them appear more morphologically distinct.

Interestingly, this Ventricleftida group also contained three other cells (TBM2, TBM3, and QCH3) that formed a distinct sub-group with no close known relatives that we call ‘Novel Clade A’ (Fig. 2). These cells are morphologically very similar to each other—dorsoventrally flattened flagellates that glide on their posterior flagellum, with no anterior flagellum visible, and a round to oblong cell shape with a median furrow that is very pronounced in the anterior part of the cell (Fig. 1Q, R, S). Apart from the somewhat generic round-oblong cell body shape, this morphology is different from other known Ventricleftida, as both *Verrucomonas* and *Ventrifissura* have two obvious emergent flagella. Some *Verrucomonas* have characteristic pigmented dimples on their surface as well, as opposed to all our cells [4], but it should be noted that Ventricleftida overall are poorly understood, with only two studies available [4, 14]. There are no environmental sequences that fall into this ventricleftid subgroup, despite the substantial amount of environmental sequence data from Cercozoa (Additional File 3: Fig. S3).

More interestingly, three other cells (TS2, SMB3, and NO2022) formed ‘Novel Clade B’ (Fig. 2), which branched with moderate support and was distant from *Micrometopion* and the newly characterized cells from the previously reported environmental NC6 group (see above; 90/74% [UFB/PMSF]). All three cells are biflagellates with oblong to ovoid cell shapes and two long, thin flagella (Fig. 1A, B, C). While the cells seem to glide on their posterior flagellum, the anterior flagellum ‘whips’ up and down along the apical 2/3 of its length, without much side-to-side movement (Videos TS2, SMB3, NO2022 — Borealis Data; we did not observe this behaviour in cell NO2022). While much more pronounced here, this behaviour is similar to that of cells in Novel Clade 6. *Micrometopion*, on the other hand, is a much smaller biflagellate that glides on its posterior flagellum and has a barely emergent anterior flagellum [3], illustrating that more detailed studies are needed to understand this group.

Similarly, cell QCF12 was sister to the newly characterized cell FiloNC4co in our multigene tree, albeit with poor support (50–60%). The SSU rRNA of the cells used in the co-assembly of FiloNC4co/’Novel Clade C’ (cells BB13, BB15, TBM7) branches with an environmental sequence (clone 9–2.2), which used to be considered a member of ‘Novel Clade 4’ previously (Additional File 3: Fig. S3; [23]) but apparently has since been viewed as a separate lineage within Cercozoa [3, 20]. In our multigene tree, FiloNC4co and QCF12 are sister to the Marimonadida in a completely different part of the tree from ‘Novel Clade 4’ (Fig. 2), representing yet another two novel uncharacterized lineages. The morphology of QCF12 shows an oblong to ovoid cell with internal granulated structure that is dorsoventrally flattened (Fig. 1D), gliding on its thin posterior flagellum with the thin anterior flagellum beating irregularly side to side (Video QCF12 — Borealis Data). Unfortunately, the SSU rRNA sequence could not be recovered from the transcriptome of cell QCF12 (which is rare, but not unheard of [25]), so we cannot rule out that QCF12 is related to representatives of any previously known environmental or characterized clade. The morphology of cells from FiloNC4co (Fig. 1E, F, G) is somewhat different from cell QCF12, with a more irregular cell shape and more pronounced side-to-side beating of the anterior flagellum in QCF12 (Videos QCF12 and BB13 — Borealis Data).

### Deeper branches

Three other cells fell in very interesting deep-branch positions: *Hedriocystis*, *Tremula* (SC1200), and co-assembly WFco (Fig. 2).

Previously, *Lapot* was characterized as part of the phylogenetically distinct Aquavolonidae (formerly Novel Clade NC10 [9, 32, 38]), Tremulida was formerly known as NC11 [39], and in SSU rRNA trees, *Tremula*, *Lapot*, and NC12 were shown to form a clade that was sister to Endomyxa [9], but without strong support. We instead recover a strongly supported clade of *Lapot*, *Tremula*, and a previously unknown lineage represented by co-assembly WFco (Fig. 2, ‘Novel Clade D’), all sister to a group consisting of Phytomyxea, Ascetosporea, and Apofilosa [40], albeit with low support. Cells in co-assembly WFCo are metabolic amoeboflagellates with two flagella (Fig. 1M, N, O, Videos WF2, WF3, WF4 — Borealis Data), suggesting some similarity to *Lapot gusevi*, which is also metabolic and possesses two unequal flagella [9]. Movement patterns of both taxa are similar as well. Furthermore, our *Tremula* SC1200 isolate is also metabolic with two emergent flagella (Fig. 1T, Video SC1200 — Borealis Data), suggesting this morphology might be ancestral to this group, but additional studies using light and electron microscopy are needed. Based on the strongly supported Tremulida + Aquavolonida + WFco (‘Novel Clade D’) clade in our multigene analyses, and the morphological similarities, it is likely that the class Skiomonadea [41] should be amended to include Aquavolonida [31] and the deeper diversity associated with this clade, particularly WFco.

Reminiscent of the historical association of Phaeodaria with Radiolaria, the order Desmothoracida in the class Granofilosea bears a strong resemblance to heliozoan amoebae (Fig. 1P) and was originally thought to be related to them; however, it is now known to be a cercozoan [39]. Granofilosea are amoeboid with distinct reticulopodia and extrusomes, and some might have a biflagellated stage in their life cycle [32]. Our cell *Hedriocystis pellucida* SC1203 represents the first molecular data for this species and the first multigene data for Desmothoracida. In the SSU rRNA tree, we recover this species branching with an environmental sequence in a clade which includes *Hedriocystis reticulata* and *Clathrulina elegans* (Additional File 3: Fig. S3), as expected. In the phylogenomic tree, *Hedriocystis* forms a deep, isolated branch within Cercozoa, as the only representative of the much larger class Granofilosea. Overall, this suggests the heliozoan-like cercozoans are likely an ancient, independent lineage much like Phaeodaria and will likely require specific attention to determine their diversity and internal evolutionary radiation.

## Conclusions

Cercozoa are a major group of eukaryotes, found abundantly in diverse ecosystems across the planet, and play key roles as primary producers, heterotrophs, and parasites [1, 8, 23]. Yet, they are dramatically understudied, with some of the sparsest genomic data in any part of the tree of life [13, 42].

**Fig. 3:**
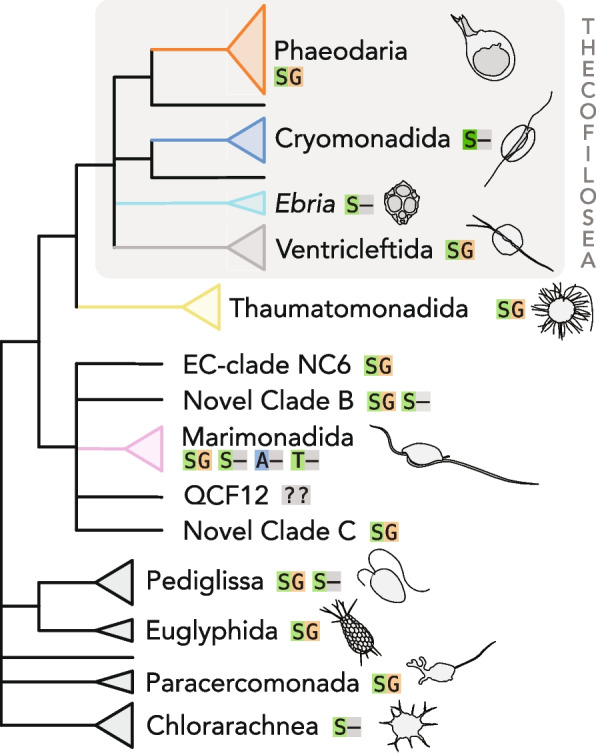
Schematic representation of the major groups of Cercozoa, with morphology sketches for each. Amino-acid insertions between polyubiquitin monomers are marked with coloured boxes next to each group

Phylogenomics has been applied to diverse members of this group relatively recently, on a small scale, based on a few available cultures [9]. Our tree roughly supports many of the conclusions of these early analyses but also massively expands on the scope of the data available (with over 3× more taxa). This analysis already goes a long way toward defining which subgroups are well-supported and which ones are not and also provides the first supported placement for a number of groups that were previously only known from a single environmental sequence or not represented by any data at all (Fig. 3).

While a full-scale analysis of character evolution is beyond the scope of this report, the tree topology and the data underpinning it should prove critical to such analyses. For example, we can already confirm that the polyubiquitin insertions that were first proposed to be a uniting character of Rhizaria [8, 31, 43–45] are indeed present in all of our transcriptomes, but interestingly, in some clades, the exact nature of the insertions varies and has a complex distribution (Fig. 3). Some cercozoan clades (Thaumatomonadida, Phaeodaria, Euglyphida, Cercomonadida, and Ventricleftida) have two amino acid insertions between polyubiquitin monomers as reported previously [31, 46], whereas others like Cryomonadida and *Ebria* only have one. A single amino acid insertion was proposed to be ancestral to Rhizaria, while a double insertion may be a more derived trait [31]. Our wealth of data complicates this view somewhat, with some shallow-branching cercozoan clades likely having secondarily lost the double amino acid insertion (e.g. Cryomonadida).

But for most of the basic biology of many of these groups, we lack enough information to really say much. As the data for Cercozoa expand, their interpretation will certainly be aided by a strongly supported tree representing all the major subgroups of Cercozoa, which appears to be technically feasible by relying heavily on the single-cell transcriptomics methods we used here, particularly considering the time and effort needed to establish cultures of protists.

## Methods

### Cell isolation and imaging

Cells were sampled from 40 different locations across North America and the Caribbean, spanning several different habitats (marine benthos, marine plankton, soil; Additional File 5: Table S1). Sampling was facilitated by the Hakai Institute (Quadra Island), the Northern Gulf of Alaska Long Term Ecological Research project (Alaska), and the Caribbean Research and Management of Biodiversity facility (CARMABI, Curaçao). For benthic samples, sediment, mud, or sand was collected from the aerated upper 2 cm. Sample extraction followed the Kimwipe method [47], with cells aggregating on coverslips after several hours. Coverslips were observed with the downside facing upwards on an inverted microscope (Leica DMIL-LED), imaged with Sony Alpha7S III or Sony Alpha7R III cameras, at either 40× or 63× magnification.

Planktonic samples were collected via an array of methods. In the Gulf of Alaska, samples were collected from the R/V Sikuliaq using a 21 µm hand net, 53 µm and 150 µm vertically towed calvets, 150 µm multinets, and Niskin bottles on CTD rosettes, from which water was concentrated using gravity filtration with a 0.8 µm (47 mm) filter (Pall). On Quadra Island, samples were collected using a hand net deployed from shore or vertical 250 µm zooplankton net tows deployed from a vessel. All samples were immediately observed after collection (or after concentration in the case of Niskin samples). Cells were imaged on an inverted microscope at 10×, 20×, 40×, or 63× magnification (Leica DMIL-LED) or a dissection microscope (Zeiss Stemi 508) with Sony Alpha7S III or Sony Alpha7R III cameras.

The single soil sample (SPO5) was isolated from forest litter, a small amount of which was submerged in dH_2_O for a day and then observed under a Leica DMIL-LED inverted microscope and imaged with a Sony Alpha7R III camera at 63× magnification.

In all cases, after imaging, cells were manually isolated from coverslips or seawater with a microcapillary, rinsed 2–6 times in drops of clean 0.2 µm filtered seawater (marine samples) or 0.2 µm filtered tapwater (soil), and dispensed into 2 µl of SmartSeq2 lysis buffer [48].

Samples SC1169, SC1200, and SC1203 were derived from crude cultures. Raw samples (Additional File 5: Table S1) were incubated at room temperature for 2 weeks with the addition of 0.025% wheat grass extract to promote bacterial growth. Cells were manually isolated with tapered Pasteur pipettes and inoculated individually into wells of a plastic 96-well plate, containing 200 μl of Pratt’s medium with 0.025% wheat grass extract. Individual wells with growing clonal cells were transferred to a Petri dish with the same medium, and the clonal cultures were further maintained by biweekly reinoculation of 1 ml of old culture into fresh medium and kept at 15 °C. Cells were imaged on Zeiss AxioVert A1 and Zeiss Axioplan 4 microscopes, the latter equipped with differential interference contrast (DIC) optics. Photographs and videos were captured on a Sony Alpha7R III camera. To increase cDNA yield due to small cell size, eight single cells of SC1169, five cells of SC1200, and five cells of SC1203 were respectively manually isolated into three tubes containing 2 µl of SmartSeq2 lysis buffer, after having been washed several times in 0.2 µm filtered Pratt’s medium.

### Single-cell transcriptomics and assembly

Isolated cells were disrupted by 2–5 freeze–thaw cycles in lysis buffer, and cDNA was generated using SmartSeq2 with 22-24 PCR cycles for cDNA amplification [48]. Libraries were prepared using Illumina DNA Prep kits for Illumina MiSeq, NextSeq 500, or NovaSeq X platforms, with 2 × 150 bp or 2 × 250 bp paired-end reads by the UBC Sequencing and Bioinformatics Consortium (Additional File 6: Table S2).

Raw reads were corrected with rcorrector version 1.0.5 [49], adapter- and quality-trimmed with Trimmomatic version 0.39 [50] using parameters ILLUMINACLIP: 2:30:10 LEADING:5 SLIDINGWINDOW:5:16 MINLEN:60, trimming adapter sequences: TSO (5′AAGCAGTGGTATCAACGCAGAGTACATGGG 3′), olido-dT (5′AAGCAGTGGTATCAACGCAGAGTACTTTTTTTTTTTTTTTTTTTTTTTTTTTTTT 3′), ISPCR (5′AAGCAGTGGTATCAACGCAGAGT 3′), Transposase1 (5′CTGTCTCTTATACACATCTCCGAGCCCACGAGAC 3′), and Transposase2_rc (5′CTGTCTCTTATACACATCTGACGCTGCCGACGA 3′). Trimmed reads were assembled with rnaSPAdes versions 3.15.5 or 3.14 with default parameters [51]. Protein-coding sequences were generated with TransDecoder version 5.5.0 [52].

### SSU rRNA phylogenetics

We extracted SSU rRNA sequences from the assemblies using barrnap version 0.9 (https://github.com/tseemann/barrnap). Extracted sequences were blasted against NCBI GenBank’s nr/nt database to identify cercozoan SSU rRNAs and potential contaminants. Cercozoan SSU rRNAs were aligned with a custom Rhizaria dataset that was constructed using previously available datasets [3, 14, 23] and supplemented with manually retrieved sequences from NCBI GenBank. After adding our new SSU rRNA sequences, the 326-taxon dataset was aligned with MAFFT E-INS-I version 7.48 [53] and trimmed with trimAl version 1.2rev59 with parameters -gt 0.9 -st 0.001 [54], yielding a 1064-bp trimmed alignment. A Maximum Likelihood (ML) analysis was carried out with RAxML-NG version 1.1.0 [55] under the GTR + GAMMA model and 1000 nonparametric bootstraps.

### Multigene phylogenetics

For our multigene dataset, we added our 119 predicted single-cell proteomes as input to PhyloFisher version 1.2.14 [56] using the default dataset. We additionally added rhizarian transcriptomes, genomes, and EST data collated in the EukProt version 3 database (https://evocellbio.com/eukprot/), euglyphid transcriptomes [57], ascetosporean and endomyxan genomes [40], a *Polymyxa betae* genome (GenBank accession GCA_003693705), three thecofilosan single-cell amplified genomes [58], and an *Orciraptor agilis* transcriptome (GenBank accession PRJEB49867).

Data were added to the database in several batches, and each time, we manually checked each of the 240 single gene trees generated as part of the PhyloFisher pipeline. Contaminant, paralogous, or otherwise aberrant sequences were deleted or omitted from the final dataset.

The final dataset consisted of 70 genes from 115 rhizarian taxa (including 7 Retaria acting as an outgroup). This dataset includes taxa that were combined into a chimeric taxon in PhyloFisher using the –chimeras flag with the select_taxa.py script. Additional File Tables S1, S2, and S3 show which taxa were subjected to this. Individual taxa used in creating these chimeras were omitted from the final tree, instead prioritizing the chimeras. Taxa and genes were chosen to maximize coverage of the single-cell transcriptomes while retaining breadth across the tree of Rhizaria. The percentages of sites and genes recovered for each taxon are reported in Additional File 7: Table S3.

A Maximum Likelihood phylogeny of the final 115-taxon, 70-gene dataset (12,261 amino acid sites) was generated using IQ-TREE2 version 2.2.0 [59] under the LG + C60 + G model with 1000 ultrafast bootstraps (UFB [60]) and under a posterior mean site frequency model (PMSF [61]) with 200 non-parametric bootstraps, using the previous LG + C60 + G tree as a guide tree.

We also generated a more taxon-comprehensive, but site-poorer phylogeny, with 128 taxa and 33 genes (4961 amino acid sites) under the LG + C60 + G model with 1000 ultrafast bootstraps.

## Supplementary Information

Additional file 1. Fig. S1. Additional light micrographs of representatives of novel cercozoan cells from which transcriptome data were obtained. Scalebars are 10 µm, except 100 µm for P1-P5, Q1-Q2, R1

Additional file 2. Fig. S2. Additional light micrographs of representatives of novel cercozoan cells from which transcriptome data were obtained. Scalebars are 10 µm, except 5 µm for I

Additional file 3. Fig. S3. Small Subunit rRNAtree of Cercozoa, showing relationships of isolated cells to known diversity. Generated with Maximum-Likelihood under the GTR + Gamma model with 1,000 non-parametric bootstraps. Sequences reported in this study in bold and red, with major clades marked. Full bootstrap supportis denoted by a circle, with supports below 50% omitted

Additional file 4. Fig. S4. Taxon-rich but gene-poor dataset. 128 taxon, 33 genes phylogeny of Rhizaria, generated under the LG + C60 + G model with 1,000 ultrafast bootstraps

Additional file 5. Table S1. Sampling information for all cells

Additional file 6. Table S2. Sequencing information for all cells

Additional file 7. Table S3. Metrics for multigene analysis

## Data Availability

Raw reads of all single cells are deposited under NCBI BioProject PRJNA1317988 and PRJNA1174372. SSU rRNA sequences are deposited under NCBI GenBank accessions PX213529-PX213634 and PX227518-PX227524. Image and video files, assembled transcriptomes, predicted proteomes, SSU rRNA alignment and tree, multigene alignment and trees, are deposited under Borealis accession10.5683/SP3/PW1ZZP.

## Abbreviations

SSU rRNA: Small subunit of ribosomal RNA
cDNA: Complementary DNA
UFB: Ultrafast bootstrap
PMSF: Posterior mean site frequency (model)
EST: Expressed sequence tag
NC: Novel clade
GTR: General time reversible (model)
