# Supplementary figures and images for: Phylogenomic tree of Cercozoa based on single-cell transcriptomes from 100 uncultured cells
Source: BMC Biol. 2026 Jan 30;24:55. doi: 10.1186/s12915-026-02536-4 (PMC12930898; doi:10.1186/s12915-026-02536-4)

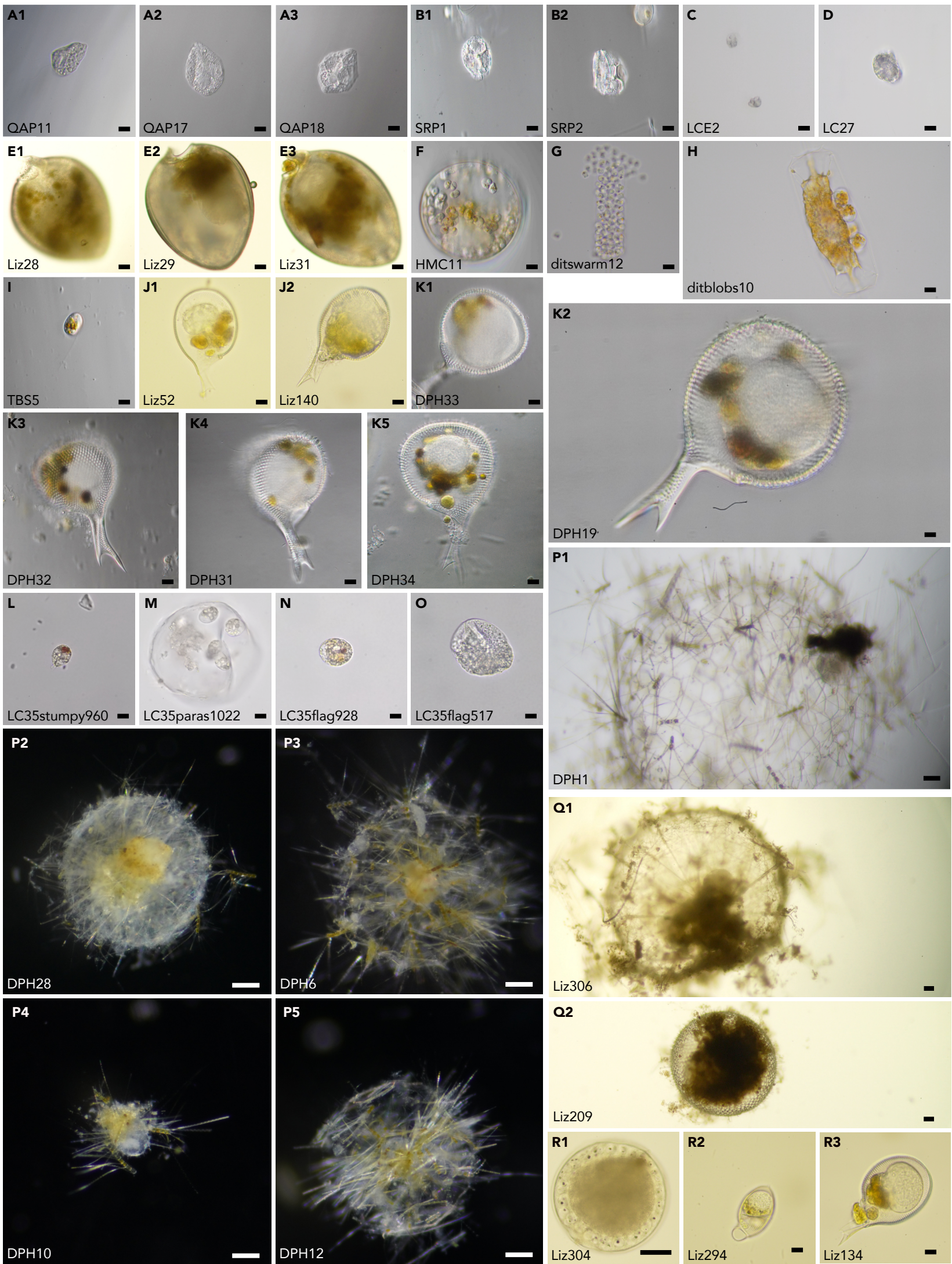

Supplement: Supplementary file 1 — Additional file 1. Fig. S1. Additional light micrographs of representatives of novel cercozoan cells from which transcriptome data were obtained. Scalebars are 10 µm, except 100 µm for P1-P5, Q1-Q2, R1 [file 12915_2026_2536_MOESM1_ESM.pdf]

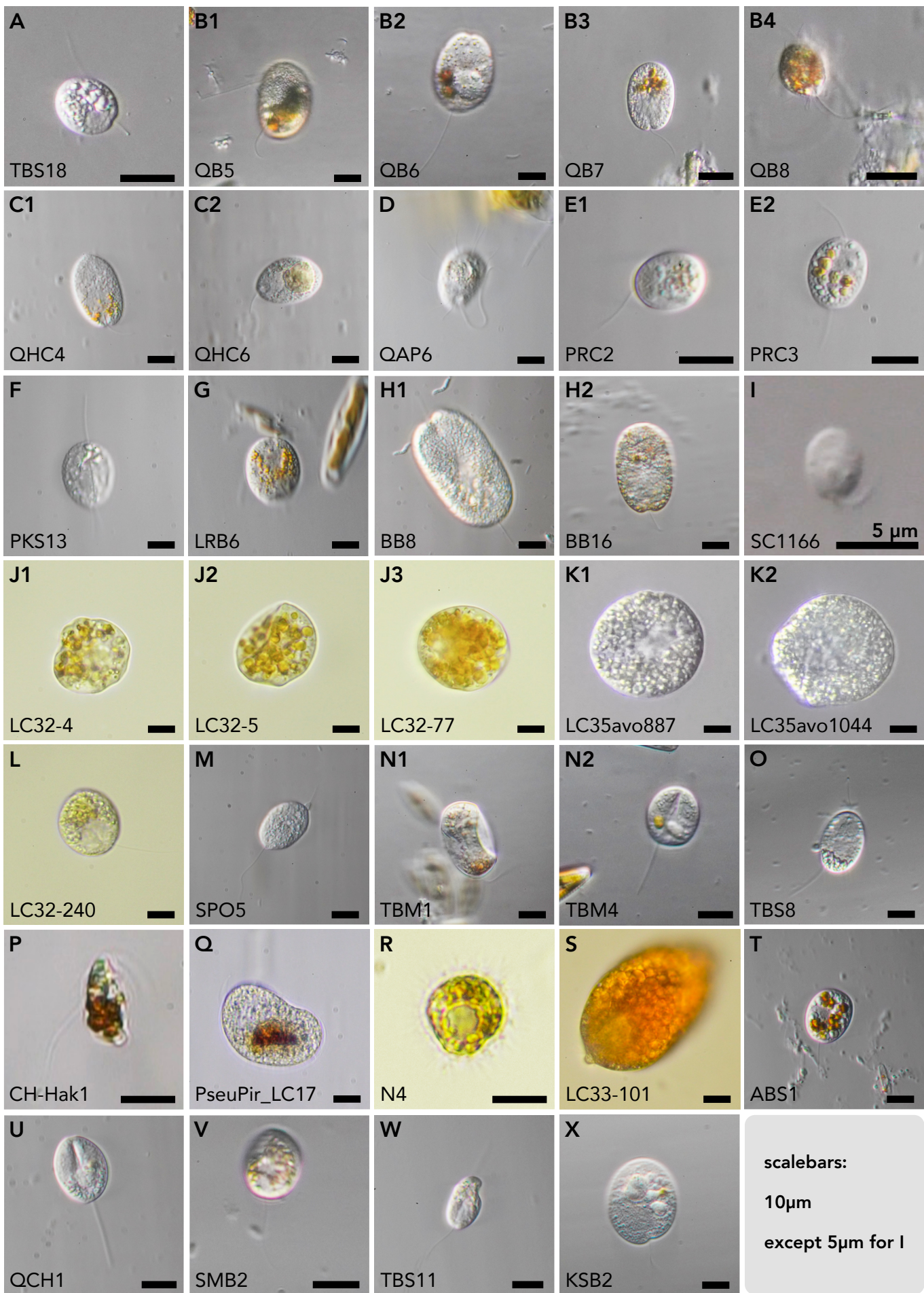

Supplement: Supplementary file 2 — Additional file 2. Fig. S2. Additional light micrographs of representatives of novel cercozoan cells from which transcriptome data were obtained. Scalebars are 10 µm, except 5 µm for I [file 12915_2026_2536_MOESM2_ESM.pdf]

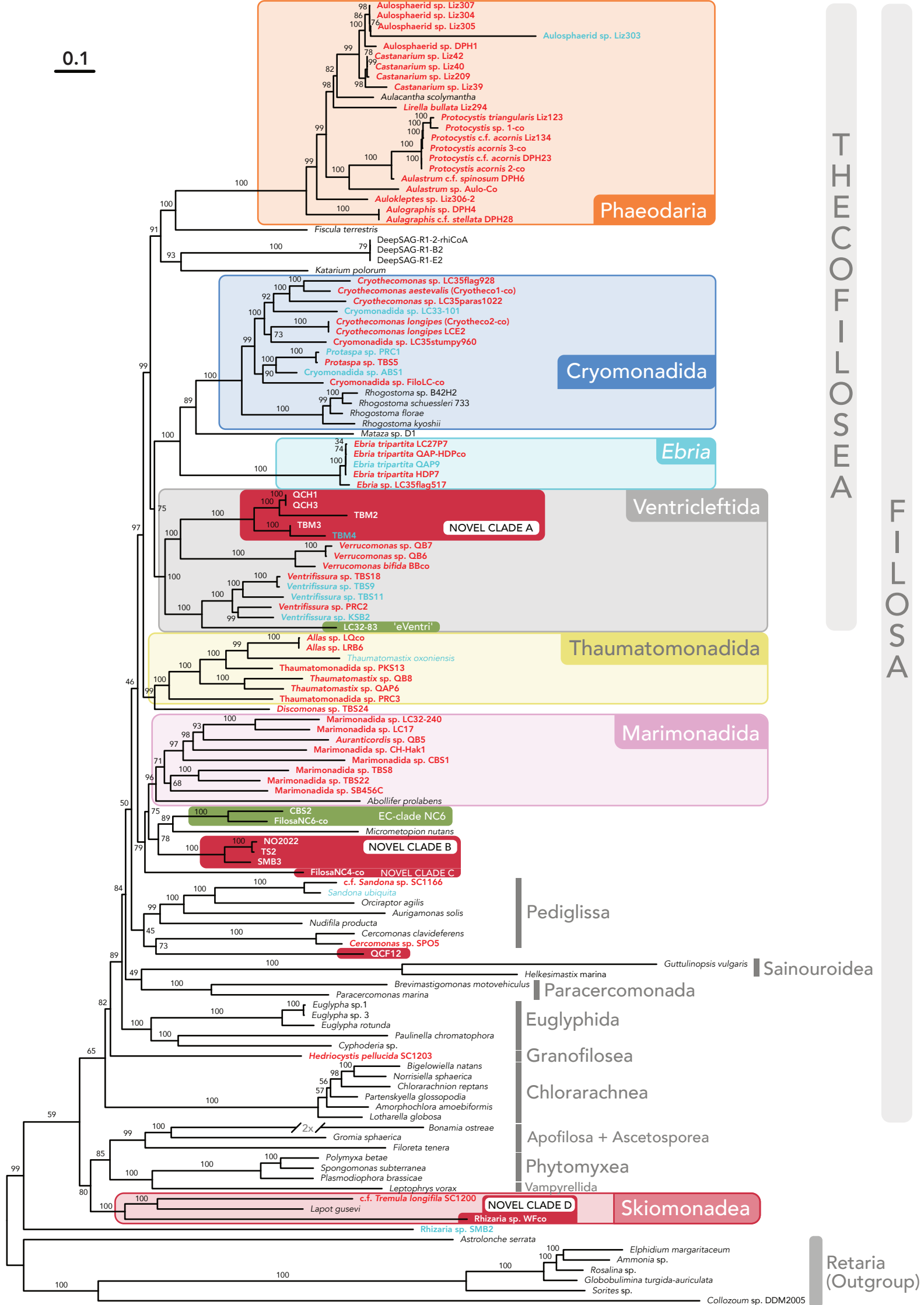

Supplement: Supplementary file 4 — Additional file 4. Fig. S4. Taxon-rich but gene-poor dataset. 128 taxon, 33 genes phylogeny of Rhizaria, generated under the LG + C60 + G model with 1,000 ultrafast bootstraps [file 12915_2026_2536_MOESM4_ESM.pdf]
