# Supplementary material for: Phylogenomic tree of Cercozoa based on single-cell transcriptomes from 100 uncultured cells
Source: BMC Biol. 2026 Jan 30;24:55. doi: 10.1186/s12915-026-02536-4 (PMC12930898; doi:10.1186/s12915-026-02536-4)
Supplement: Supplementary file 3 — Additional file 3. Fig. S3. Small Subunit rRNAtree of Cercozoa, showing relationships of isolated cells to known diversity. Generated with Maximum-Likelihood under the GTR + Gamma model with 1,000 non-parametric bootstraps. Sequences reported in this study in bold and red, with major clades marked. Full bootstrap supportis denoted by a circle, with supports below 50% omitted [file 12915_2026_2536_MOESM3_ESM.pdf]

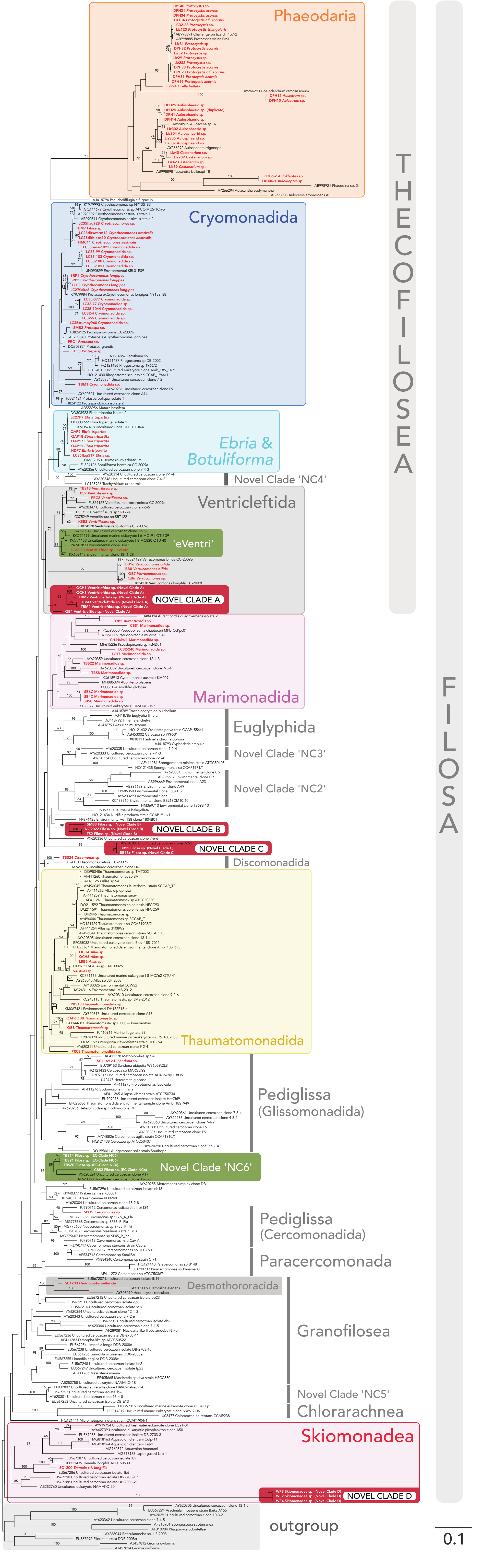

Phaeodaria
Cryomonadida
Ebria & Botuliforma
Novel Clade 'NC4'
Ventricleftida
'eVentri'
NOVEL CLADE A
Marimonadida
Euglyphida
Novel Clade 'NC3'
Novel Clade 'NC2'
NOVEL CLADE B
NOVEL CLADE C
Discomonadida
Thaumatomonadida
Pediglissa (Glissomonadida)
Novel Clade 'NC6'
Pediglissa (Cercomonadida)
Paracercomonada
Desmothoracida
Granofilosea
Novel Clade 'NC5'
Chlorarachnea
Skiomonadea
NOVEL CLADE D
outgroup
THECOFILOSEA
FILOSA
0.1
